# Frequent food insecurity among injection drug users: correlates and concerns

**DOI:** 10.1186/1471-2458-12-1058

**Published:** 2012-12-08

**Authors:** Carol Strike, Katherine Rudzinski, Jessica Patterson, Margaret Millson

**Affiliations:** 1 Dalla Lana School of Public Health, University of Toronto, 155 College Street, Toronto, ON, M5T 3M7, Canada; 2 Centre for Addiction and Mental Health (CAMH), Toronto, ON, Canada

**Keywords:** Food insecurity, Injection drug use, Sharing injection equipment, HIV risk behaviour

## Abstract

**Background:**

Food insecurity and nutrition are two topics that are under-researched among injection drug users (IDUs). Our study examined the extent and correlates of food insecurity among a sample of IDUs and explored whether there is an association between food insecurity and injection-related HIV risk.

**Methods:**

A cross-sectional survey was conducted using interviewer-administered questionnaires. Data were collected at a needle exchange program in London, Ontario, Canada between September 2006 and January 2007. Participants included 144 English-speaking IDUs who had injected drugs in the past 30 days. Participants were asked about their socio-demographic characteristics, HIV risk behaviours, food insecurity, and health/social service use.

**Results:**

In the past 6 months, 54.5% of participants reported that on a daily/weekly basis they did not have enough to eat because of a lack of money, while 22.1% reported this type of food insecurity on a monthly basis. Moreover, 60.4% and 24.3% reported that they did not eat the quality or quantity of food they wanted on a daily/weekly or a monthly basis, respectively. Participants reported re-using someone else’s injection equipment: 21% re-used a needle, 19% re-used water, and 37.3% re-used a cooker. The odds of sharing injection equipment were increased for food insecure individuals.

**Conclusions:**

Findings show that IDUs have frequent and variable experiences of food insecurity and these experiences are strongly correlated with sharing of injection-related equipment. Such behaviours may increase the likelihood of HIV and HCV transmission in this population. Addressing food-related needs among IDUs is urgently needed.

## Background

Food insecurity is most common among people with low-income and unstable housing, persons who are unemployed/on welfare, and individuals with mental illness and/or a history of illicit drug use [1-3]. Most research about food insecurity in Canada focuses on low-income households and HIV-positive individuals, meaning much less is known about the food experiences of other marginalized populations such as the urban poor or people who inject drugs (IDUs) [4,5].

Food insecurity is defined as an inability to acquire or consume a sufficient quantity of food or an adequate quality of food in socially acceptable ways, or the uncertainty of being able to do so [6,7]. In other words, food insecurity is an apprehension about and/or the reality of going hungry due to the lack of access or means to acquire food [8]. Hunger is the most extreme consequence of food insecurity and allows for the differentiation between moderate and severe forms of this concept [9,10]. An adequate diet is an essential predictor of the health and nutritional status of a population [7,11]. Food security – or the ready availability and accessibility of enough nutritionally adequate food for an active, healthy life – is a universal dimension of household and individual well-being [12,13].

In Canada, food insecurity is typically measured for the general public through various national health surveys (i.e., National Population Health Survey, Canadian Community Health Survey) [14]. Recent findings estimate that 2.7 million Canadians, or 8.8% of the population, live in food insecure households [15]. Although food insecurity does not immediately endanger life, it does lead to diminished physical and mental health status, and influences the social, psychological, and daily functioning of those individuals who experience it [2,12]. This condition has been associated with serious health problems in the general population – including obesity, diabetes, heart disease, hypertension, and depression [16-19]. Canadians living with food insecurity are more likely than those who are food secure to rate their health as poor or fair [7].

Injection drug use is associated with many negative physical, emotional, social, economic and legal outcomes, including: elevated risks of acquiring HIV, Hepatitis C virus (HCV), and sexually transmitted infections (STIs); injection-related injuries to skin and veins; violence and victimization; fatal and non-fatal overdose; depression and other mental health problems; social isolation; poor educational attainment; homelessness, unemployment and poverty; frequent incarceration; and, involvement in the sex trade [20-30]. Given the high rates of poverty, homelessness, and unemployment among IDUs it might be expected that these individuals would also have tenuous access to a stable source of food and poor diets, yet there is limited research on this key determinant of health, especially in resource-rich or developed countries [5,8].

The limited research evidence currently available does show that between 30% and 70% of drug-using individuals report some level of food insecurity [1,31-35]. One Canadian study of an HIV-positive population found that among individuals who reported ever having injected drugs, 33.7% were food insecure and 43.7% were severely food insecure (with hunger) [10]. A more recent study of HIV-negative IDUs in Vancouver, Canada found that 64.7% of participants reported being hungry and unable to afford enough food [5]. Several studies specifically showed that rates of food insecurity tend to be higher among individuals addicted to drugs [2,32,36]. A complex set of pathways at the individual (i.e., behavioural risk of acquisition and HIV morbidity and mortality), household (i.e., insufficient quality and quantity of food, anxiety, deprivation and alienation, poor coping), and structural (ecological, poverty, education, gender and stigma) levels helps us to understand the link between food insecurity and HIV/AIDS [37]^.^ At the individual and household level, drug users are often said to be at an increased risk of food insecurity due to their chaotic lifestyles, unstable housing and limited finances, as well as their previous and ongoing health problems [31]. For IDUs, the competing demands of addiction and subsistence are a daily struggle, wherein eating a nutritionally adequate diet may not always be a priority [38]. ‘Drug binges’ – patterns of intense drug use often lasting for days at a time during which food, sleep, and basic hygiene are neglected – are common among users of stimulant drugs in particular and significantly affect nutritional health and dietary intake [38-40]. In addition, drug addiction has been shown to modify eating habits, often causing individuals to adopt poor dietary patterns such as an irregular eating schedule, eating fewer meals per week, skipping meals, fasting to prolong the effects of drugs, eating late at night, and eating alone [38,41,42].

Issues of homelessness or unstable housing, common among IDUs, result in a lack of food preparation and storage facilities [43,44]. Meanwhile, the limited finances of IDUs, which are primarily spent on maintaining the drug habit, again hinder access to groceries and food selection [31]. As a result, fast, cheap, and easy-to-prepare foods are the primary components of IDUs’ diets, contributing to poor nutrient intake [32,43,44]. In fact, drug users have been found to have lower dietary intakes of fruits, vegetables, grain and dairy products, while consuming more high-fat sweets and salty snack foods [2,32,33,45]. Subsequently, studies show that IDUs are more prone to vitamin deficiencies (including vitamin A, C, and E, as well as iron, thiamin and calcium), anemia, malnutrition with observable emaciation, lower body mass index (BMI), gastrointestinal distress, tooth decay, and decreased appetite than non-drug users [31,32,44,46].

Existing studies of IDUs indicate a heightened risk for infectious diseases, such as tuberculosis, HIV and HCV, which combined with chronic drug use can severely compromise nutritional status [36]. The relationship between food insecurity and HIV is the most developed in the literature with current findings showing that, for HIV-infected individuals suffering from food insecurity, problems include: reduced adherence to highly active anti-retroviral treatment (HAART) as well as reduced treatment efficacy; lower BMI; severe wasting; lower CD4 count; lower odds of viral suppression; and a high risk of mortality [1,8,32,47-49]. Evidence shows that food insecurity is 5 to 7 times greater among people living with HIV than the Canadian average [8,10,47]. Most studies on food insecurity and nutritional deficiencies among IDUs tend to focus on HIV-positive populations – where self-reported hunger has been correlated with unprotected sex and risk of HIV transmission [8,48]. Since nutritional deficiencies caused by food insecurity have been shown to increase viral load and reduce treatment efficacy in HIV-infected IDUs, the risk of transmission through behaviours such as re-use of needles and injection equipment would also be elevated, yet this has not been well researched [8,50,51].

In Canada, as in other Western countries, food insecurity emerged as a serious social and public health problem at a time of declining public spending on social welfare and shifting economic policies [2,4]. Structural drivers such as no universal national programs for nutritionally vulnerable populations, like IDUs, likely influence the prevalence of food insecurity amongst this population. Current responses include implementation of many ad hoc local food programs (i.e., food banks, soup kitchens, etc.), the success of which is difficult to ascertain [11,52]. Yet, food security is considered a basic human right under several covenants of international law and food insecure individuals should not have to resort to emergency food supplies, begging, stealing and/or scavenging for food [2].

Taking into consideration the research gaps identified above and keeping in mind that food security is a fundamentally important determinant of health that affects most aspects of daily life, our study strived to examine the prevalence of food insecurity among people who inject drugs and to explore if this experience is correlated with risky injection behaviours linked with the transmission of HIV and other blood-borne infections. Specifically, we asked the following questions: how often do IDUs report being food insecure; are some IDUs more likely than others to report food insecurity; and is food insecurity correlated with injection-related risk behaviours such as sharing injection equipment (i.e., needles, water, cookers, and/or filters)?

## Methods

Data were collected for a larger study examining the relationships between HIV risk behaviours, drug treatment readiness, and basic needs. For this study, we recruited IDUs in London, Ontario from September 2006 to January 2007 to participate in a cross-sectional survey. London is located 150km from Toronto, Ontario with a population of approximately 352,000. The IDU Outreach Coordinator and Community Co-Investigators based at the Counterpoint Needle Exchange Program (NEP) in London advertised the study by word of mouth and printed flyers.

IDUs expressing an interest in the study were introduced to the research staff who explained the study’s purpose. Those with a desire to participate in the research protocol were screened to determine their eligibility: 16 years of age or older; English speaking; and having injected in the past 30 days. IDUs deemed eligible were told about the study requirements and compensation ($20) in more detail and then asked for informed consent. Participants who were too intoxicated to give informed consent were asked to return on another day. This study was approved by the Research Ethics Board at the University of Toronto and the Centre for Addiction and Mental Health.

In the absence of a sampling frame, this study employed a stratified, quota sampling technique to maximize the representativeness of the sample in terms of gender in relation to the local IDU population (i.e., 70% male and 30% female). Participants were recruited until the quota in each stratum was reached. Using a structured, interviewer-administered, questionnaire, participants were asked about their socio-demographic characteristics, injection and sexual risk behaviours, perceived social support, drug treatment readiness, program satisfaction, housing status, income and employment, as well as their experiences of food insecurity and health/social service use. In addition, participants completed the self-report version of the Addiction Severity Index [50]. We modified the three food insecurity questions from the Canadian Community Health Survey 1.1 [10] to reflect the 6-month recall period common to most other questions on our survey. Using the same question structure, we added a fourth question to enquire about lack of food because of a ‘drug binge’.

To characterize the prevalence of food insecurity and examine correlates of injection risk behaviours, we used univariate and bivariate statistical tests and logistic regression. The analyses were performed using SPSS statistical software package version 20 (SPSS Inc., an IBM Company, Chicago, IL, USA) and conducted in two steps. To develop the logistic regression models, chi-squared tests were used to examine the strength of the associations between each of the dependent variables –re-use of needles, re-use of cookers, re-use of mixing/rinsing water, and re-use of filters – and the independent variables that have been previously identified as related to re-use of injection equipment [51]. The independent variables included: age (under versus over 25 years); housing instability (i.e., number of moves); stayed/slept outdoors in past 6 months; injected opiates, cocaine or crack cocaine in past 6 months; injected drugs outdoors in past 6 months; need assistance to inject; injected alone; self-reported HCV and HIV status; Addiction Severity Index composite score; and Addiction Severity psychiatric composite score. In light of our interest in food insecurity, we also included the variable - reporting not enough to eat on a daily/weekly or monthly basis in past 6 months because of a lack of money - to the regression analyses. We used only one of the food insecurity variables for this analysis for three reasons. First, correlations between each of the five variables related to food insecurity (i.e., worry about enough to eat because of a lack of money, did not have enough to eat because of a lack of money, did not eat the quality of food wanted because of a lack of money, did not eat or drink water because of a drug binge, and used a food program) all exceeded 0.555 and were statistically significant. Second, including all five variables in the logistic regressions would introduce collinearity and over-estimate effects. Third, in light of the highly correlated nature of the variables, we examined bivariate analyses comparing each dependent variable (i.e., re-use of needles, re-use of cookers, re-use of mixing/rinsing water, and re-use of filters) with each food insecurity variable (i.e., worry about enough to eat because of a lack of money, did not have enough to eat because of a lack of money, did not eat the quality of food wanted because of a lack of money, did not eat or drink water because of a drug binge, and used a food program). We selected the variable ‘reporting not enough to eat on a daily/weekly or monthly basis in past 6 months because of a lack of money’ because unlike the other food insecurity variables, it was significantly associated with each of the injection equipment re-use variables. We conducted analyses separately for each piece of equipment. To build the models, we used standard criteria where independent variables in the bivariate analyses reaching significance of ≤ 0.250 (these analyses are not shown) were entered into the logistic regression model [53]. These variables included: age under 25 years versus 25 years and over, injected outdoors in the past 6 months, self-reported HCV status positive versus negative, injected opiates in the past 6 months, and injected crack cocaine in the past 6 months. We report only associations found to be significant. Also, we report the Hosmer Lemeshow test for each logistic regression [54]. When this test is insignificant, it indicates that there is not a significant difference between the observed and predicted values and thus the model is a good fit.

## Results

Of the 144 IDUs we recruited, 72.2% were male, over half had never married (54%), more than half (53%) had not finished high school, and only a small percentage (5.6%) were employed (see Table 1). In the past 6 months, the average income for study participants, from all potential sources, was approximately $4000. When asked about their mood in the 30 days prior to the survey, 55% reported feeling depressed and/or hopeless. Over half of participants had slept outdoors in the last 6 months (51%) and many had moved, some multiple times, in that time span (54%). Injection of crack cocaine and opiates (illicit and prescription) were very common (≥80%), and more than three-quarters of the sample reported having injected outside in the last 6 months. Based on self-report data, 3% of participants were HIV-positive and 53% were HCV-positive.

**Table 1:** Demographic characteristics and injection risk behaviours over the past 6 months

	
Male	72.2%
Never married	54%
Completed < high school	53%
Employed full or part time	5.6%
Not employed or disabled	86.1%
Self-employed	7.6%
Average 6 month income all sources	$4,000
Depressed/hopeless past 30days	55%
Moves 0 to 2 times	54%
Injected crack	80%
Injected opiates	83%
Injected outside	78%
Slept outdoors	51%
HIV positive (self-reported)	3%
Hepatitis C positive (self-reported)	53%
Re-used a needle	21%
Reused water	19%
Reused a cooker	37%
Re-used a filter	18%

Overall, 54.5% of participants reported that on a daily/weekly basis they did not eat enough because of a lack of money, 52.4% reported that on a daily/weekly basis they worried that there would not be enough food and 60.4% did not daily/weekly eat the quality or variety of foods they wanted (see Table 2). More than half of the participants (57.6%) reported not eating or drinking water because of a ‘drug binge’ (i.e., extended drug use). Other participants experienced less frequent food insecurity and on a monthly basis 22.1% did not eat enough because of a lack of money; 24.3% claimed that they worried there would not be enough food; and 24.3% did not eat the quality or variety of foods they wanted. A ‘drug binge’ interrupted eating and drinking for 24.3% of the sample on a monthly basis.

**Table 2:** Food insecurity by gender, age, injected outdoors, self-reported HCV status, injected opiates and injected cocaine

	**Did you worry that there would not be enough to eat because of a lack of money?**					**Did you not have enough to eat because of a lack of money?**					**Did you not eat the quality or variety of foods that you wanted because of a lack of money?**				
	**Daily/Weekly**	**Monthly**	**Never**	***X***^**2**^	**p**	**Daily/Weekly**	**Monthly**	**Never**	***X***^**2**^	**p**	**Daily/Weekly**	**Monthly**	**Never**	***X***^**2**^	**p**
Total	52.4	24.8	22.8			54.5	22.1	23.4			60.4	24.3	15.3		
Gender															
Men	50.5	22.9	26.7		ns	53.3	21.0	25.7		ns	59.6	21.2	19.2		ns
Women	57.5	30.0	12.5			57.5	25.0	17.5			62.5	32.5	5.0		
Age															
<25	60.0	13.3	26.7		ns	53.3	13.3	13.3		ns	53.3	13.3	13.3		ns
≥ 25	51.5	26.2	22.3			54.6	23.1	22.3		ns	61.2	25.6	13.2		ns
Injected outdoors															
Yes	55.9	20.7	23.4		ns	55.9	17.1	27.0	9.160	.001	62.2	20.7	17.1		ns
No	38.7	41.9	19.4			45.2	41.9	12.9			51.6	38.7	9.7		
Self- reported HCV status															
-ve	54.1	23.0	23.0		ns	52.5	16.4	31.1		ns	57.4	21.3	21.2		
+ve	50.7	26.1	23.2			52.2	29.0	18.8			59.4	30.4	10.1		
Injected opiates in the past 6 months															
Yes	58.3	20.8	20.8		ns	58.3	12.5	29.2		ns	66.7	12.5	20.8		ns
No	51.7	25.8	22.5			53.3	24.2	22.5			59.2	26.7	14.2		
Injected crack cocaine in the past 6 months															
Yes	47.8	28.3	23.9		ns	45.7	58.2	30.4		ns	50.0	32.6	17.4		ns
No	55.1	23.5	21.4			23.9	21.4	20.4			65.3	20.4	14.3		
	**Did you not eat or drink water because of a ‘drug binge’ (i.e., extended drug use)?**					**Did you use a food program?**									
	Daily/Weekly	Monthly	Never	*X*^2^	p	Daily/Weekly	Monthly	Never	*X*^2^	p					
Total	57.6	24.3	18.1			33.3	52.1	14.6							
Gender															
Men	57.7	25.0	17.3	6.596	.037	30.8	50.0	19.2	6.593	.037					
Women	57.5	22.5	20.0			40.0	57.5	2.5							
Age															
<25	60.0	26.7	13.3		ns	40.0	57.5	2.5	6.337	.042					
≥ 25	40.0	26.7	33.3		ns	32.6	55.0	12.4							
Injected outdoors															
Yes	55.9	17.1	9.160	27.0	.001	36.0	46.8	17.1	7.377	.025					
No	45.2	41.9	12.9			19.4	74.2	6.5							
Self- reported HCV status															
-ve	59.0	24.6	16.4		ns	49.3	31.0	19.7		ns					
+ve	55.1	23.5	21.7			46.1	43.8	10.1							
Injected opiates in the past 6 months															
Yes	41.7	29.2	29.2		ns	60.8	23.3	15.8		ns					
No	41.7	54.2	4.2			31.7	51.7	16.7							
Injected crack cocaine in the past 6 months															
Yes	52.2	23.9	23.9		ns	60.2	24.5	15.3	ns						
No	30.4	47.8	21.7			34.7	54.1	11.2							

Inquiries about the use of food programs revealed that IDUs used these services, with 33.3% of the sample reporting daily/weekly use and 52.1% reporting monthly use. Examining these results by gender and age, women and younger IDUs were more likely to use food programs (e.g., food banks, soup kitchens, lunch and dinner programs) on both a daily/weekly basis and a monthly basis than men. Moreover, men were much more likely to claim to have never used a food program than women.

Many participants reported re-using someone else’s injection equipment. Specifically, 21% re-used a needle, 19% re-used water, 37.3% re-used a cooker, and 18% re-used a filter. Results from the logistic regressions that examined the associations between re-use of needles, cookers, mixing/rinsing water, and filters, and independent variables are shown in Table 3. Overall, food insecurity was associated with an increased prevalence of the risk behaviours, while being HCV positive was associated with decreased prevalence of each of the risk behaviours. The odds of re-using needles were increased for those reporting food insecurity (OR=2.7), and decreased for HCV-positive status (OR=0.341) and under age 25 (OR=0.87). The odds for cooker re-use were increased for IDUs reporting food insecurity (OR=1.9) and decreased for HCV-positive status (OR=0.445). For sharing water, the odds were increased for those reporting opiate injecting (OR=7.0) and food insecurity (OR=2.6), and decreased for HCV-positive status (OR=0.311). For filter re-use, the odds were increased for those reporting food insecurity (OR=3.1) and decreased for HCV positive status (OR=0.265).

**Table 3:** Logistic regression results for each dependent variable: re-use of needles, re-use of cookers, re-use of filters and re-use of filters

**Independent variables**	Adjusted ORs*				**Hosmer Lemoshow Test**	
	**AOR**	**95% C.I**		**p**	***χ***^**2**^**(df)**	**p**
**Logistic regression 1: Re-use of needles**						
Age ≥ 25 years	.280	.087	.902	.033	4.157 (6)	ns
Food insecure	2.743	1.056	7.126	.038		
HCV +ve	0.341	0.127	0.913	.032		
**Logistic regression 2: Re-use of cookers**						
HCV +ve	0.445	.206	.961	.039	1.566 (4)	ns
Food insecure	1.904	1.023	4.078	.049		
**Logistic regression 3: Re-use of mixing/rinsing water**						
Food insecure	2.591	.982	6.837	.054	0.479 (4)	ns
HCV +ve	0.311	.115	.838	.021		
Injected opiates	7.021	0.857	57.517	.069		
**Logistic regression 4: Re-use of filters**						
Food insecure	3.112	1.082	8.956	.035	2.930 (5)	ns
HCV +ve	0.265	.088	.792	.017		

* Based on bivariate analyses, we entered the following independent variables into the regression analyses - age under 25 years versus 25 years and over, injected outdoors in the past 6 months, self-reported Hepatitis C status positive versus negative, injected opiates in the past 6 months, injected crack cocaine in the past six months and food insecure (i.e., did not have enough to eat because of a lack of money - and above show only variables found to be significant in the logistic regressions. Adjusted odds ratios refer to the contribution of each independent variable after controlling for the contribution of other significant, independent variables in the logistic regression model.

## Discussion

In comparison with other Canadians, our data suggest that IDUs are between 2.5 and 6 times more likely to report experiencing food insecurity, when considering monthly (22.1%) and daily/weekly (54.5%) instances during which participants did not have enough to eat due to a lack of money. In 2004, the Canadian Community Health Survey estimated 8.8% of Canadians experienced food insecurity in the previous 12 months [14]. While 8.8% is likely an underestimate, the difference between IDUs and other Canadians is vast.

It is difficult to compare instances of food insecurity between various studies because there is no standard measure for individuals. Many studies adapt questions from national health surveys or the Radimer/Cornell scale, which consider food insecurity at the household level [7,10,13]. Even when the frequency (i.e., daily vs. monthly) and level (i.e., moderate vs. severe) of food insecurity is considered, it often differs from study to study.

In our study, we considered instances of food insecurity experienced daily/weekly or monthly. In this way we were able to show that IDUs have frequent and variable experiences of food insecurity. Severe food insecurity (with hunger) ranged from one-fifth (22.1%) of the sample, who experienced this monthly, to just over half of participants (54.5%), who experienced this on a daily/weekly basis. Two studies, concerned with similar drug user populations, found a slightly higher level of severe food insecurity in the last month (~64%) as compared to our study [5,33]. In our study, the mild/moderate range of food insecurity (worry about sufficient food; 24.8% monthly to 52.4% daily/weekly) is remarkably similar to the range of severe food insecurity (with hunger) (22.1% monthly to 54.5% daily/weekly). Normen *et al.*[10] and Weiser *et al.*[49] found mild/moderate food insecurity to be 33.7% and 24%, respectively, in their drug user populations, coinciding with the lower end of our range. In addition, our study considered not only the quantity of food available to IDUs, but also the quality and variety – giving us information about another aspect of the food insecurity experiences of this population.

There is also a strong association between food insecurity and adverse mental health issues. Experiencing symptoms of depression has been associated with a higher incidence of food insecurity across a range of populations [5,35,47,49]. One study found that self-reported hunger was independently associated with experiencing symptoms of depression in the past week (67%) [5]. This finding was similar to the rate, over the last 30 days, of feelings of depression and hopelessness (55%) in our study. However, it is unclear if depression is the cause or consequence of food insecurity. Food insecurity can also lead to thiamine and iron deficiency and contribute to problems like apathy, anxiety, irritability, and depression [55]. Research also shows that 1 in 5 people with mental illness, including depression, report problems with finding adequate food [56].

It has been suggested that food insecurity may contribute to unsafe injection practices if food insecurity interferes with access to harm reduction programs and other health and social support programs [49]. Since we collected data from a population accessing a needle and syringe program, it is difficult to assess this relationship. However, our data and existing research do show an important link between injection drug use and access to food programs. Despite the fact that IDUs are at an increased risk of experiencing food insecurity and may be more in need of food-related assistance, people who have drug problems attend charity food supply programs significantly less than people who do not have such problems. Kaufman *et al.*[2] found that drug users (14%) used food assistance programs more than 2 times less than non-drug users (39%). Reasons for this may include a lack of awareness or information among people with drug problems about the availability of food services provided, a preference for managing one’s poverty in private, and drug treatment service personnel not knowing how to address the problem of food insecurity or experience of stigma that discourage attendance at programs [2,40,52]. In our study, a moderate percentage (33.3%) of IDUs used food programs on a daily/weekly basis, while about half (52.1%) of the sample used these services on a monthly basis. Interestingly, when analysing the results by gender our study revealed that women were more likely to use food programs on a daily/weekly and monthly basis whereas men were more likely to report never using food programs. Our findings diverge from previous research which shows that male drug users have dinner from a food program more frequently whereas females have more snack meals [33]. Other studies report that female drug users, on average, have poorer nutritional statuses than males [33,57] In our study, participants were asked about food programs specifically, thus those who receive meals through shelters or other organizations (i.e., detention centers, prison, churches, etc.) may have overlooked these sources when responding. However, male IDUs are typically more likely to reside in shelters and/or to have been incarcerated, thus their access to food could be improved through these sources. In light of this, there is a need to consider the different strategies and barriers that men and women face when dealing with food insecurity [38]^.^ In addition, IDUs’ high reliance on others for food can place these individuals in exploitive relationships where they can be coerced into a variety of dangerous behaviours (e.g., survival sex) in order to secure this precious commodity [4,44].

Research links markers of poverty and behavioural risk to severe food insecurity. Severe food insecurity is independently correlated with a 2.5-fold elevated proportional odds of engaging in unprotected vaginal or anal sex, and HIV-positive women are 2 times as likely as HIV-positive men to engage in unprotected sex [8]. Our study uniquely considered the association between food insecurity and sharing injection equipment. The findings show that the odds of sharing injection equipment (i.e., needles, water, cookers, and filters) were increased for food insecure individuals. This is a significant finding in that sharing injection equipment increases the odds of transmitting or acquiring HIV [58-61] which, in the environment of food insecurity, can lead to even more pronounced health problems. Essentially, food insecurity and HIV risk may be linked in a complex and bi-directional cycle that may contribute to more rapid progression of HIV [62]. Taking into consideration the much higher rate of self-reported HCV infection in our study population, it becomes important to consider what these findings mean for the transmission of other blood-borne diseases through sharing injection equipment among food insecure IDUs. In addition, more research is needed to determine if and how the pathways between HIV and food insecurity are similar and/or different between HCV and food insecurity. Since HCV, like HIV, has the potential to alter nutritional status significantly – i.e., patients with HCV lose weight, develop anemia, neutropenia, and have liver problems leading to dietary intolerance or limited nutrient intake – HCV in combination with food insecurity can also have detrimental results with respect to treatment, disease progression, and health complications [48].

Interpretation of our findings should be done with consideration of the limitations. First, lack of a sampling frame prevented random sampling. However, the data were collected using a stratified convenience sampling method to ensure that the sample reflected the gender division within this population. In terms of the age distribution of the population, anecdotal evidence from the NEP suggests that our sample reflects that of the local IDU population. Our findings were corroborated by the IDU co-investigators on our team who noted that the findings were consistent with their personal experiences and their knowledge of drug-using behaviours and food insecurity in the local IDU community. Second, our data are based on self-report which can be open to recall and social desirability biases. However, self-report data from similar populations of illicit drug users have proven valid [63,64]. Third, our data are cross-sectional and we could not assess the directionality of the relationship between food insecurity and injection risk behaviours.

## Conclusions

Our research shows that the experiences of food insecurity among a population of IDUs are both frequent and variable and that these experiences are associated with higher odds of sharing injection equipment. In light of this, future programs need to incorporate targeted food assistance strategies for IDUs, as well as supportive housing models and safer equipment distribution. In addition, routine assessment for food insecurity should be incorporated into treatment and prevention programs for IDUs to monitor food insecurity more closely in this at-risk population. Service providers also need to carefully consider the nutritional status, lifestyle characteristics, and socioeconomic problems that can compromise IDUs’ access to food and dietary intake and increase their risk of acquiring a blood-borne infection. However, more livelihood interventions are needed to address the upstream drivers of food insecurity and drug dependence.

## Competing interests

The authors declare that they have no competing interests.

## Authors’ contributions

CS conceived of the study, led its design and coordination, and drafted and finalized the manuscript. KR drafted and finalized the manuscript. JP assisted with analyses and drafting of the manuscript. PM conceived of the study, participated in its design and coordination, assisted with the analyses, and helped to draft the manuscript. All authors read and approved the final manuscript.

## Pre-publication history

The pre-publication history for this paper can be accessed here:

http://www.biomedcentral.com/1471-2458/12/1058/prepub
